# Mechanism of WS_2_ Nanotube Formation Revealed by *in Situ*/*ex Situ* Imaging

**DOI:** 10.1021/acsnano.4c01150

**Published:** 2024-05-03

**Authors:** Vojtěch Kundrát, Libor Novák, Kristýna Bukvišová, Jakub Zálešák, Eva Kolíbalová, Rita Rosentsveig, M.B. Sreedhara, Hila Shalom, Lena Yadgarov, Alla Zak, Miroslav Kolíbal, Reshef Tenne

**Affiliations:** a Department of Molecular Chemistry and Materials Science, Weizmann Institute of Science, Rehovot 7610001, Israel; b Thermo Fisher Scientific, Vlastimila Pecha 12, 62700 Brno, Czech Republic; c Central European Institute of Technology, Brno University of Technology, Purkyňova 123, 61200 Brno, Czech Republic; d Chemistry and Physics of Materials, University of Salzburg, Jakob-Haringer-Strasse 2A, 5020 Salzburg, Austria; e Solid State and Structural Chemistry Unit, Indian Institute of Science, CV Raman Road, Bangalore 560012, India; f Department of Chemical Engineering, Ariel University, Ariel 4070814, Israel; g Faculty of Science, Holon Institute of Technology, Golomb Street 52, Holon 5810201, Israel; h Institute of Physical Engineering, Faculty of Mechanical Engineering, Brno University of Technology, Technická 2, 616 69 Brno, Czech Republic

**Keywords:** WS_2_ nanotube, sulfidation, *in situ*, *ex situ*, electron microscopy, reaction mechanism

## Abstract

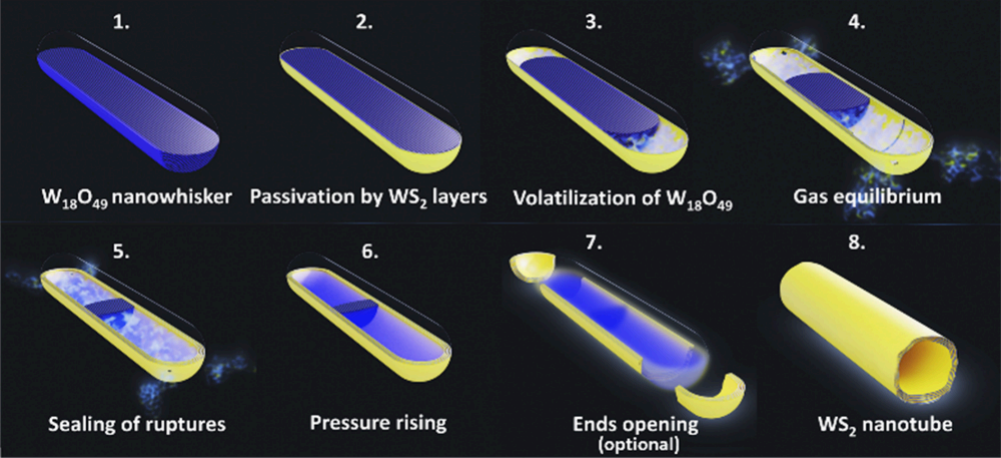

Multiwall WS_2_ nanotubes have been synthesized from W_18_O_49_ nanowhiskers in substantial amounts for more than a decade. The established growth model is based on the “surface-inward” mechanism, whereby the high-temperature reaction with H_2_S starts on the nanowhisker surface, and the oxide-to-sulfide conversion progresses inward until hollow-core multiwall WS_2_ nanotubes are obtained. In the present work, an upgraded *in situ* SEM μReactor with H_2_ and H_2_S sources has been conceived to study the growth mechanism in detail. A hitherto undescribed growth mechanism, named “receding oxide core”, which complements the “surface-inward” model, is observed and kinetically evaluated. Initially, the nanowhisker is passivated by several WS_2_ layers via the surface-inward reaction. At this point, the diffusion of H_2_S through the already existing outer layers becomes exceedingly sluggish, and the surface-inward reaction is slowed down appreciably. Subsequently, the tungsten suboxide core is anisotropically volatilized within the core close to its tips. The oxide vapors within the core lead to its partial out-diffusion, partially forming a cavity that expands with reaction time. Additionally, the oxide vapors react with the internalized H_2_S gas, forming fresh WS_2_ layers in the cavity of the nascent nanotube. The rate of the receding oxide core mode increases with temperatures above 900 °C. The growth of nanotubes in the atmospheric pressure flow reactor is carried out as well, showing that the proposed growth model (receding oxide core) is also relevant under regular reaction parameters. The current study comprehensively explains the WS_2_ nanotube growth mechanism, combining the known model with contemporary insight.

## Introduction

Transition metal dichalcogenide (TMDC) nanotubes (NTs), especially those composed of WS_2_ and MoS_2_, have been known for over three decades as the subject of both experimental and computational studies.^1^ The first mass-produced synthesis of pure WS_2_ nanotubes quantities was described in ref (2). Typically, these multiwall nanotubes have diameters ranging from 20 to 150 nm and are 10–50 μm long. Multiwall MoS_2_ nanotubes grown via the chemical vapor transport technique were described more than two decades ago.^3^ Various synthetic strategies for WS_2_ nanotubes were described in the literature.^4−12^ Recently, submillimeter long WS_2_ nanotubes exhibiting aspect ratios of >3000 were reported.^13^ Optical and electrical characteristics of WS_2_ have recently received a considerable amount of attention. It was found, for example, that they display a superconducting transition at 5.8 K when using ionic liquid gating.^14^ A strong bulk photovoltaic effect in these nanotubes was observed, intimately linked to the inherent breaking of inversion symmetry and violation of time-reversal symmetry by the chiral nanostructures.^15^ Other notable findings include the pronounced coupling between optical cavity modes and excitons in MoS_2_ ^16^ and WS_2_ ^17^ nanotubes, the manifestation of second harmonic generation,^18^ realizations of a torsional resonator,^19^ and the sliding ferroelectricity.^20^ The last was utilized to store optical data in a nanotube array and subsequently access this information. Furthermore, when incorporated into polymers in trace amounts, WS_2_ nanotubes significantly bolstered their strength and fracture toughness against impact, which could have immense impact on, among others, medical technologies and 3D printing.^21−23^

The growth mechanism of WS_2_ nanotubes through sulfidation of WO_3–*x*_ (0 ≤ *x* ≤ 0.18) nanoparticles at elevated temperatures (>800 °C) was discussed extensively in the literature.^2,4,5,10^ A slight reduction of the WO_3–*x*_ nanoparticles leads to the fast growth of W_18_O_49_ nanowhiskers through a volatile phase.^2,24^ The evaporation of the tungsten oxide was shown to be greatly facilitated by water molecules, which are formed during the reductive conversion of tungsten oxide to tungsten sulfide. The water molecules recombine with the solid oxide forming volatile WO_3–*x*_·H_2_O.^25−27^ Alternatively, the volatile tungsten oxide has been proposed as cyclic cluster W_4_O_11_,^10,28^ which is formed as a result of the reduction of the oxide precursor by H_2_.

Subsequently, a reaction of the tungsten oxide nanowhiskers with H_2_S and H_2_ gases takes place, leading eventually to the formation of WS_2_ NTs via the so-called “surface-inward” mechanism.^2,3^ According to this two-step growth model of the nanotubes, first, H_2_S gas reacts rapidly (<1 min) with the oxide nanowhisker surface, forming a few (2–4) closed WS_2_ layers on top of the oxide core. Subsequently, a slow diffusion of hydrogen sulfide inward and oxygen outward occurs, somewhat akin to the Kirkendall effect.^29−31^ The slow quasi-epitaxial layer-by-layer inward growth of the WS_2_ layers gradually consumes the oxide core, ultimately resulting in a hollow nanotube. The advent of high-resolution electron microscopy and *in situ* electron microscopy provides detailed insight into the growth mechanism of such nanotubes, which is the topic of the present work.

Scanning and high-resolution transmission electron microscopy (SEM and HRTEM) play a pivotal role in the structural characterization of nanomaterials.^32−37^*In situ* SEM and TEM reactions have been studied in the past.^38−42^ However, reactive gases like H_2_S have been rarely used in these reactions, especially at elevated temperatures, where the genuine metallic parts of the microscope are under permanent threat during the reaction, not to mention the exceptional toxicity of the gas. The μReactor allows *in situ* observation of high-temperature heterogeneous reactions within a scanning electron microscope.^43,44^ This reactor was exploited recently to study the growth mechanism of W_18_O_49_ nanowhiskers under a hydrogen atmosphere.^44^ These tungsten oxide nanowhiskers have been investigated extensively in the past.^45,46^ They grow along the *b*-axis [010] direction and crystallize in a monoclinic symmetry (P2/*m*, JCPDS no. 84-1516) with lattice constants *a* = 18.31(2), *b* = 3.839(8), *c* = 14.00(1) Å and β = 115.19(9)°.^47^ The highly anisotropic growth rate along the *b*-axis is a testimony to the high surface energy of the {010} surface.

In the current work, an expedient SEM with a mounted μReactor was retrofitted for the *in situ* observation of sulfidation reactions. For that purpose, H_2_S and H_2_ sources were added, which permitted the study of the high-temperature conversion of W_18_O_49_ nanowhiskers into hollow multiwall WS_2_ nanotubes. In the following text, it is shown that a complementary and hitherto unknown growth mechanism takes place during the synthesis of multiwalled WS_2_ nanotubes.

The growth mechanism of the WS_2_ nanotubes is discussed by considering the growth environment in the μReactor and compared with atmospheric pressure reactions. Three fundamental questions are addressed in this work:

- What is the WS_2_ nanotube formation mechanism by the sulfidation of W_18_O_49_ nanowhiskers?
- What are the key parameters influencing the morphology of the nanotubes?
- Are the nanotubes and insight gained by the μReactor sulfidation relevant to the atmospheric pressure reaction in the flow reactor which is used for the growth of a pure phase of WS_2_ nanotubes in large quantities?

**Figure 1:**
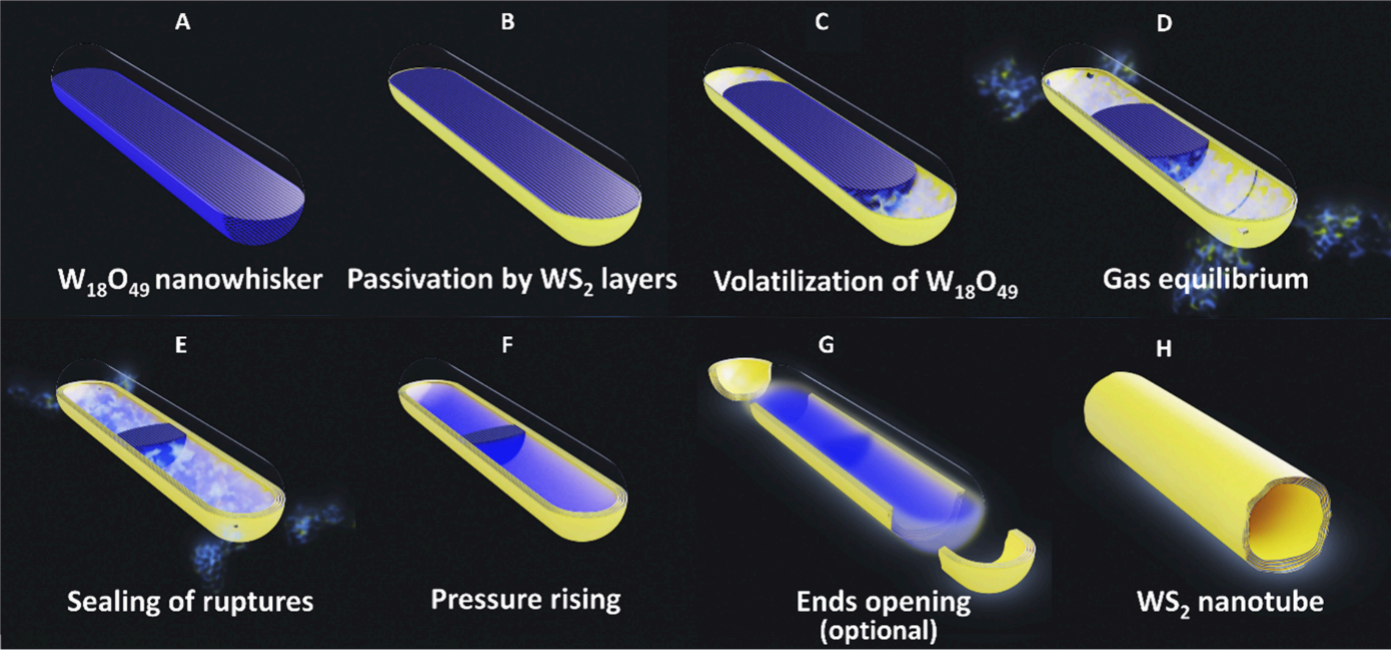
Scheme of the growth mechanism for WS_2_ nanotube formation. The figure is formed from the selected frames of Video 1. Panels A–G display midsection models of the nascent nanotube, while panel H shows projection of a completed nanotube.

In this work, a previously unknown growth mechanism of WS_2_ nanotubes, i.e., the “receding oxide core”, is discussed. This growth mechanism complements the well-established “surface inward” pathway.^2,3^ It is furthermore shown that under certain circumstances this growth mode can predominate after the first few WS_2_ layers have been formed on the nanowhisker surface by the surface-inward mechanism. In Figure 1 and related Video 1, the growth mechanism of WS_2_ nanotubes from tungsten suboxide nanowhiskers is foreshadowed as a graphical scheme. There are several stages of nascent nanotube formation. Initially, the tungsten suboxide nanowhisker (Figure 1A) is passivated by several WS_2_ layers (Figure 1B) via the “surface inward” reaction, producing the edifice of the nascent nanotube. Meanwhile, the heated tungsten suboxide core is evaporated from its tips within the WS_2_ shell (Figure 1C). The evaporating oxide expands partially, escaping through cracks and defects in the WS_2_ walls (Figure 1D). Simultaneously, the reactive H_2_S and H_2_ gases diffuse into the empty space in the core and react with the volatilized tungsten oxide. Further, WS_2_ layers are deposited internally from the vapor phase within the cavity, allowing sealing of cracks and defects in the multilayer wall (Figure 1E). The residual oxide core continues to evaporate, increasing the inner gas pressure (Figure 1F). In some cases, the pressurized vapors break through the weakest spots, opening the WS_2_ nanotube at the end (Figure 1G,H). Hints of this mechanism are scattered in a number of studies by various research groups published throughout the years. However, a comprehensive understanding of it was lacking hitherto.

The bottom line of this study is that an unexplored growth mechanism of the nanotubes from tungsten oxide nanowhiskers is observed. Generally speaking, the so-called “receding oxide core” mechanism operates in conjunction with the well-established “surface inward” sulfidation and is critical for the growth of the tungsten disulfide nanotubes. Ergo, the tungsten suboxide nanowhiskers react with H_2_ and H_2_S gases in both heterogeneous vapor–solid and homogeneous vapor phase reactions. Gaining this insight is crucial for understanding the nuances of the well-known synthesis of WS_2_ nanotubes, adding this synthetic pathway to the canon of growth methods for TMDCs in general.

## Results and Discussion

**Figure 2:**
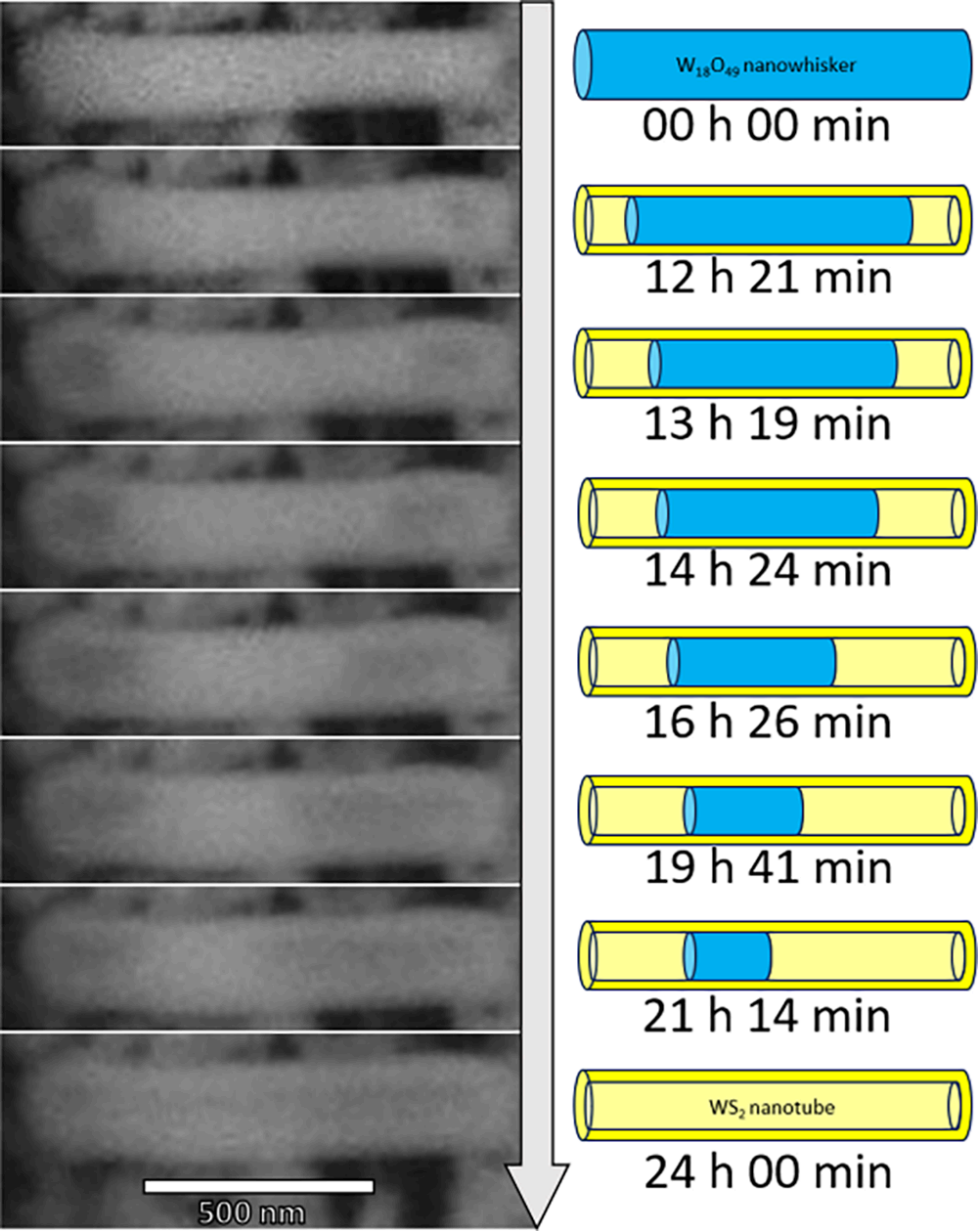
Selected images from the SEM sequence of the *in situ* observed sulfidation reaction of W_18_O_49_ nanowhisker at 900 °C in H_2_S (50 Pa) and H_2_ (25 Pa), Video 2. A graphical scheme replicating the progress of the SEM sequence is displayed on the right. At the beginning of the reaction (00:00), the W_18_O_49_ nanowhisker appeared as a bright monolith in the center of the figure. After more than 12 hours (12:21), nascent nanotube cavities appeared at the ends of the whisker (covered with invisible layers of WS_2_), which are enlarged with time. The oxide core diminished along the ⟨010⟩ axis, as shown graphically. The darker cavities gradually expanded from the tips of the nanowhisker toward its middle (13:19 → 21:14). After 24 h (24:00), the reaction was completed, the bright tungsten suboxide core disappeared, and a completely hollow WS_2_ nanotube was formed.

A preliminary experiment was performed by SEM and observed continually. Using the upgraded μReactor, the sulfidation of W_18_O_49_ nanowhiskers at 900 °C was observed *in situ*, as shown in Video 2 and related Figure 2, which consisted of selected series of SEM images from the measured sequence. Notably, morphological changes in the nanowhisker core could be delineated by operating the electron beam at 20 keV and detecting backscattered electrons. Notwithstanding the limited resolution, a clear progression of a cavity forming and growing axially in the center of the nanowhisker could be detected. At the beginning of the reaction (Figure 2, 00:00), the nanowhisker (approximately 140 nm thick and 820 nm long) appeared as a bright monolithic structure. Unfortunately, the swift conversion of the oxide surface into closed WS_2_ layers is not detectable in the SEM. The passivation of the tungsten suboxide whisker surface by several WS_2_ layers delays the reaction within the inner core, which becomes apparent only after 12 hours when the nanotube cavity observed as a darker contrast emerges (Figure 2, 12:21) at the specimen ends. The backscattered signal and its brightness difference indicated a hollow character of the forming WS_2_ nanotube (Figure 2, 12:21 and on). The contrast of the tungsten oxide in the core of what used to be a monolithic nanowhisker diminishes gradually with time until, after 24 h, the bright core fully disappears, and the nanotube is entirely hollow (see Figure 2, 24:00). Visibly, the cavity forges ahead from both edges of the nanowhisker, however asymmetrically with the right-hand side (rhs) progressing faster than the left-hand side. As will be understood from the discussion below, this difference can be attributed mostly to the partial pressure of the tungsten oxide vapor in the cavity, which is determined to a large extent by the perfectness of the WS_2_ tip. Apparently, the tip at the rhs of the nanowhisker is less perfect and leaky compared to the left one. Hence the oxide vapors are released faster, and the W_18_O_49_ core disappears faster on that side.

A similar sequence of *in situ* sulfidation steps of another nanowhisker is shown in Figure S1. The reaction was performed at 800 °C. This experiment took much longer to complete (almost 47 h) than the one shown in Figure 2 due to the lower reaction temperature, the more considerable length, and the diameter of the present nanowhisker. The observed Video 2 and the related Figure 2 shows that the growth mechanism of the WS_2_ nanotube is different from the previously presumed diffusion path in the flow reactor.

**Figure 3:**
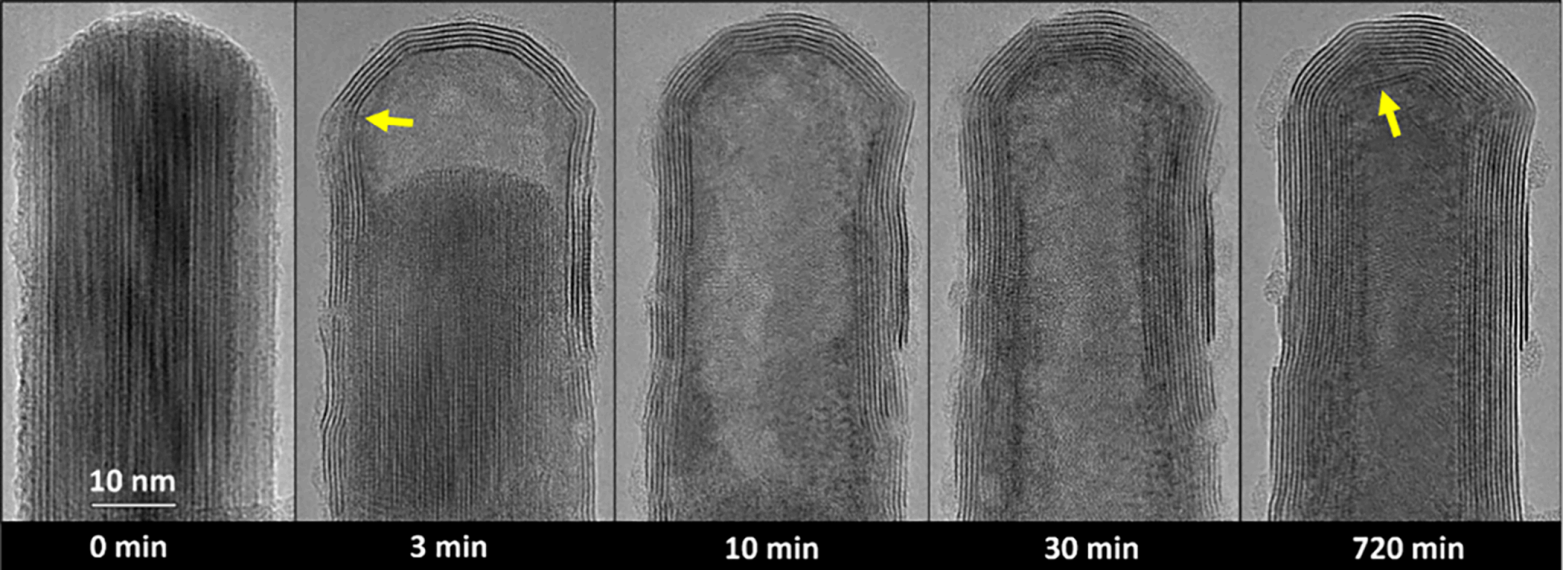
*Exsitu* TEM images sequence of the sulfidation of W_18_O_49_ nanowhisker at 1000 °C in H_2_S/H_2_ (50 + 25 Pa). The pristine W_18_O_49_ nanowhisker (before the reaction) is shown (0 min). Reaction in the μReactor (within the SEM) was interrupted at preselected times (3, 10, 30, and 720 min) for the *ex situ* TEM measurements. The yellow arrow after 3 min of reaction indicates the position of a significant defect in the WS_2_ wall. The yellow arrow after 720 min reaction indicates the gap between the two innermost WS_2_ layers at the tip and the outer ones formed earlier.

In order to obtain a more detailed view of the process, an *ex situ* sequential experiment was performed in the μReactor, and the specific tube was periodically transferred and analyzed at preselected times in the TEM (Figure 3). For these experiments, fresh W_18_O_49_ nanowhiskers were drop-cast on the MEMS chip. TEM analysis of a selected pristine nanowhisker was executed (Figure 3, 0 min). The TEM-analyzed nanowhisker was approximately 30 nm thick with a round and symmetric tip and was more than 500 nm in length. The uniform grayish pattern indicated a single domain and homogeneous profile of the nanowhisker. The nanowhisker specimen was subjected to *in situ* sulfidation in the SEM for 3 min at 1000 °C in a mixture of hydrogen and hydrogen sulfide gas and subsequently analyzed again by TEM (Figure 3, 3 min). The reaction time was already sufficient for forming 3–5 WS_2_ layers on the surface of the W_18_O_49_ nanowhisker. The so-called passivation WS_2_ layer is clearly formed via a “surface-inward” mechanism. The H_2_S, together with H_2_, penetrated the surface of the W_18_O_49_ nanowhisker and created several WS_2_ layers enveloping the remaining oxide core. Continually, the gases diffused through the layers, mainly through defects, forming additional WS_2_ layers beneath the existing ones. Therefore, the W_18_O_49_ core was partially consumed by the sulfidation reaction. In parallel, the oxide core vaporizes, preferentially at its ends, forming a cavity at the tip of the specimen. The volatilization of tungsten oxide nanowhiskers from its tips is a well-studied phenomenon, observed even *in situ* in TEM, as part of the known evaporation-growth mechanism of tungsten suboxides.^24,44,48,49^ A similar evaporation occurs within the nascent nanotube, as indicated by the formation of the cavities (Figures 2 and 3). Interestingly, the curved WS_2_ layers at the tip contain many structural defects and stacking faults (Figure 3, 3 min). Such defects hasten the diffusion of hydrogen and H_2_S through the passivating WS_2_ layers into the nanotube core. Moreover, a part of the tungsten suboxide within the core and near the tip was vaporized, increasing the pressure inside the cavity. The WO_2_(OH)_2_ ^21−23^ vapors breached the WS_2_ wall through a significant defect (see the yellow arrow), increasing the size of the cavity at the tip. The sample was then moved back to the μReactor, and the sulfidation reaction was continued for another seven min, after which it was stopped, and the TEM analysis was repeated (Figure 3, 10 min). The tungsten oxide core continued shrinking- receding from the tip and expanding, thereby, the cavity. Surprisingly, more WS_2_ layers were deposited within the cavity on the already existing WS_2_ walls, even on the tip, with no direct contact with the tungsten suboxide mass. Further sequential measurement of the sulfidation reaction confirmed this observation. This clearly indicates that the formation of WS_2_ layers goes through the deposition from the vapor phase within the cavity. The nanotube cavities are formed from the tips of the nascent nanotube, subjected to the highly anisotropic evaporation of the oxide core. This evaporation provides reactant WO_2_(OH)_2_ gas, which, together with present H_2_S and H_2_, creates a fertile mixture for reaction and deposition of WS_2_ layers from the vapor phase within the hollow core. At 30 min of reaction time, the TEM analysis revealed that the oxide had receded from the nanotube tip and was already out of view (Figure 3, 30 min). Meanwhile, another three to four WS_2_ layers were deposited on the inner wall, yet the layers were still not free of defects. Finally, after 12 h (Figure 3, 720 min), the nanotube exhibited highly oriented and almost perfect walls at the tip. Interestingly, the two innermost layers at the tip were misaligned with the outer layers, forming minuscule hollow space (see yellow arrow). The process of sulfidation of the whole W_18_O_49_ nanowhisker was complementary observed by *ex situ* in TEM (Figure S2). The TEM sequence shows the progress of the cavities from the two ends of the nascent nanotube.

**Figure 4:**
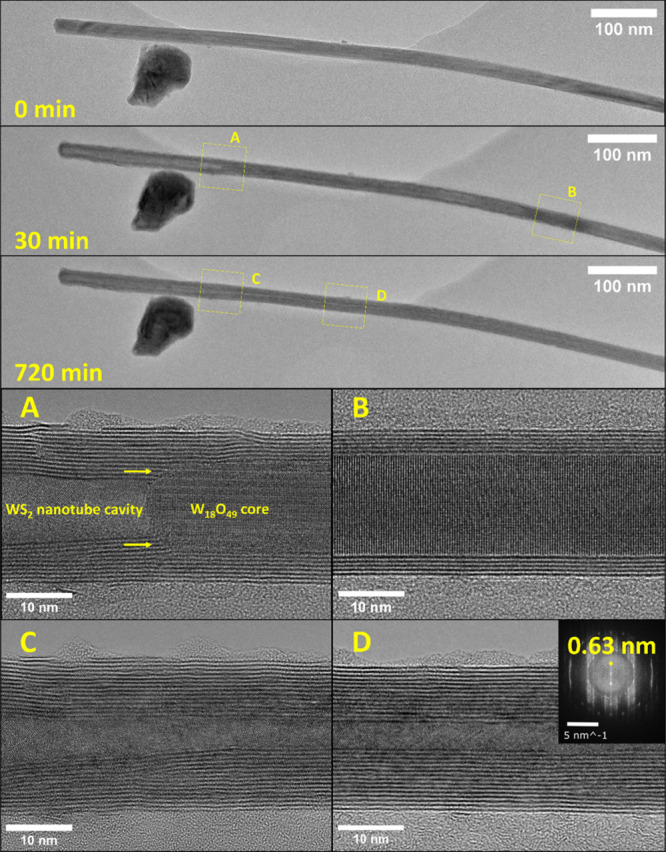
*Ex situ* TEM observation of the nanotube formation with diminishing nanotube cavity at 1000 °C in H_2_S/H_2_ (50 + 25 Pa). A long, relatively thin nanowhisker was preselected on the MEMS chip (0 min). TEM analysis of the sulfidation after 30 and 720 min shows the progress of the nanotube cavities from the tips of the nascent structure. (A) displays the interface between the receding oxide core, forming nanotube cavity, and the WS_2_ layers. (B) shows the passivated oxide core with 4–5 WS_2_ layers formed by the “surface-inward” mechanism. (C) and (D) are detailed observations of the WS_2_ nanotube formed after 12 h reaction in the μReactor. The inset in (D) shows Fast Fourier transform (FFT) of the TEM picture with marked interlayer distance 0.63 nm corresponding to WS_2_ nanotubes.^13^ The amorphous phase on the surface of the nanotube is carbon contamination formed after exposure to the electron beam. Most likely, the source of the surface contamination was the solvent (isopropanol) during sample preparation or from the transport between the SEM and the TEM.

Figure 4 shows a TEM analysis of a sequence of another nanotube formed in the μReactor (at 1000 °C) and analyzed *ex situ* in the TEM. While Figure 3 describes the local reaction environment near the tip, Figure 4 displays the overall picture mainly in the nanotube center. The reaction started with a long W_18_O_49_ nanowhisker (0 min). After 30 min of reaction, the oxide core diminished, in the same manner as in Figures 2 and 3, while a cavity was formed in the nanotube. In the magnified zone (A), one can observe the new WS_2_ layers growing right from the oxide core as well as the thickening of the wall at the oxide-sulfide interface beneath the receding oxide surface. The WS_2_ layers grow directly from the edges of the tip of the tungsten oxide core (yellow arrows). This is most probably also due to the character of the oxide evaporation, which could be described as an oscillatory vapor–solid mass transport.^24^ The mechanism of the deposition of tungsten oxide nanowhisker was studied earlier in detail,^24^ showing that the exact spot of the process happens on the edge of the tip. Close observation of the nascent WS_2_ nanotube shows the growth of new WS_2_ layers from the very edge of the oxide tip (Figure 4A, marked by yellow arrows). The growing layers adhere to the preexisting ones. The magnified image (Figure 4B) exhibits perfect passivation of the oxide core by 4–5 WS_2_ layers. These layers are clearly formed by the “surface-inward” reaction mechanism. The kinetics of the “surface-inward” mechanism are affected by both the gas pressure in the reactor and the number of defects in the passivation layer. The passivation layers limit the gas inlet to the nonevaporated core. Nevertheless, the inflow of reactive gases persists, through both the cavity of the nanotube and the space between its layers. After 12 h of reaction, the oxide core has reacted completely, leaving a pure WS_2_ nanotube. In panels (Figure 4C&D), magnified TEM images of the diminishing cavity diameter away from the tip and toward the center of the nanotube are shown. Especially in panel D, the nanotube cavity is already decently small yet well aligned. The Fourier transform (inset of Figure 4D) clearly reveals the *c*/2 interlayer spacing (0.63 nm) between the WS_2_ layers in the nanotube.

Incidentally, simultaneously with the transformation of the W_18_O_49_ nanowhisker into a WS_2_ nanotube, shown in Figure 4, a W_18_O_49_ nanoparticle near the left tip of the nanowhisker is transformed into a fullerene-like (IF) WS_2_ structure that is clearly observed. Notwithstanding the poor contrast, it is clearly observed that while the oxide core shrinks with time, more WS_2_ layers add up in the core until a hollow IF nanoparticle is formed. The transformation of WO_3_ into IF-WS_2_ nanoparticles follows the same mechanism as before, i.e., through surface-inward reaction.^50,51^

The evaporation of the oxide from the core outside leads to a violation of the stoichiometry between the tungsten oxide nanowhisker and the tungsten disulfide nanotube. In other words, some portion of the tungsten atoms of the nanowhisker are transformed into the walls of a nascent WS_2_ nanotube, and the rest evaporates out. This distinction highlights the significant role of evaporation in determining the cavity dimensions. This phenomenon is visualized in Figures 4 and S3, where the nanotube cavity’s diameter is larger near the tip than at the center. Presumably, the evaporation rate of the oxide near the tip is high. At the same time, in the middle of the nanotube, the reaction is almost stoichiometric; hence, the cavity’s diameter is significantly smaller.

**Figure 5:**
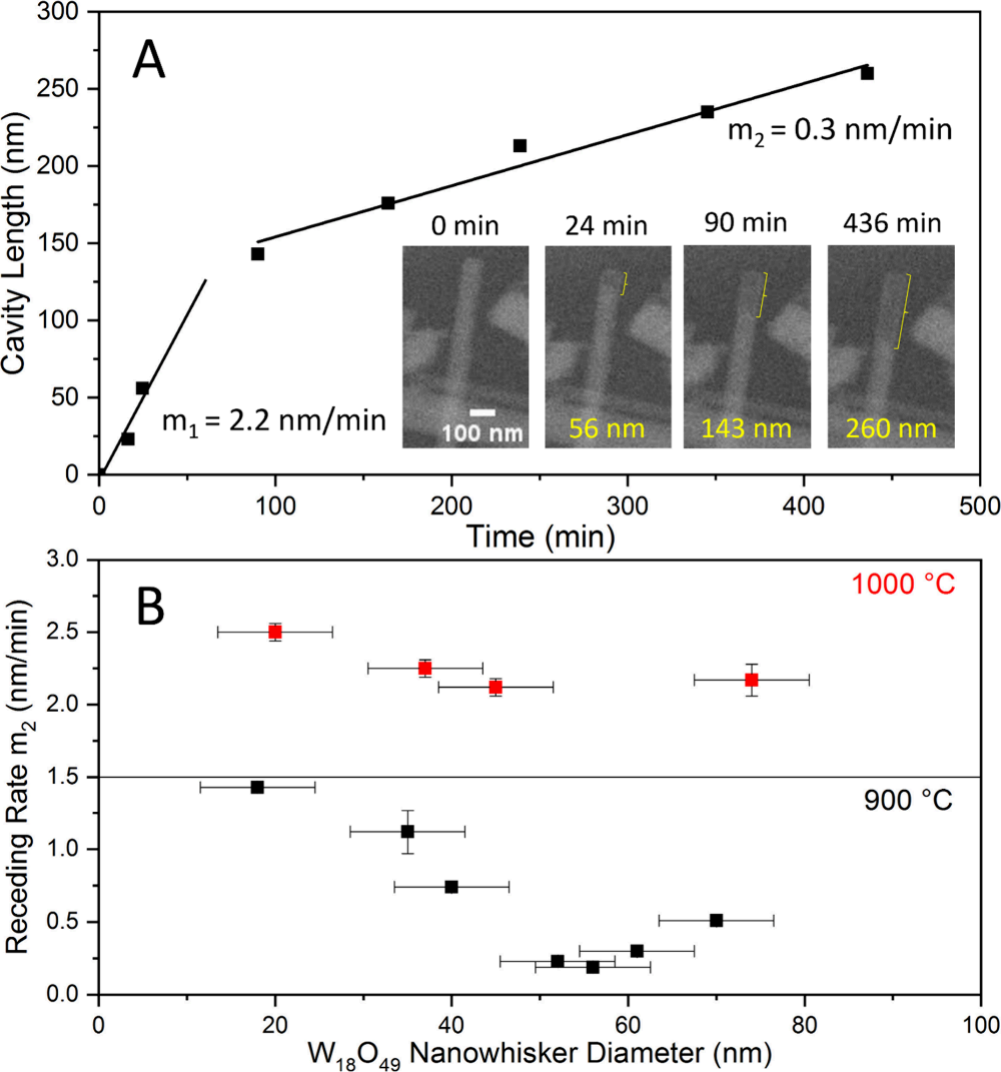
(A) Kinetic rate of the receding oxide within the nascent WS_2_ nanotube. The initial cavity formation rate (*m*_1_ = 2.2 nm/min) is more than seven times higher than that in the later phase of the reaction (*m*_2_ = 0.3 nm/min). (B) Dependence of the rate of cavity formation/receding oxide as a function of the diameter of the tungsten oxide nanowhisker and the reaction temperature.

The *in situ* SEM observations allowed to study the time dependence of the oxide core’ receding within the nascent nanotube. The kinetics of the reaction (Figure 5A) were constructed from the exemplary case displayed in *in situ* SEM Video 3. The receding of the oxide core along the <010> direction and formation of the nanotube cavity was observed at 900 °C. Interestingly, the rate of receding-oxide *m* was relatively swift in the initial phase of the reaction (*m*_1_ = 2.2 nm/min). Later, the process slowed down significantly (*m*_2_ = 0.3 nm/min). The reaction continued further on until the whole nanowhisker was turned into a nanotube. The behavior presented in Figure 5A is similar for all of the monitored nanowhiskers and confirms the insight gained in Figure 4, i.e., the oxide evaporation rate is faster at the tip of the forming tube and gets slower as the reaction progresses deeper into the core. Furtherly, the kinetic study focused on the rate of receding of the oxide core at two reaction temperatures (900 and 1000 °C) and for variable nanowhisker diameters (Figure 5B). In order to construct Figure 5B, kinetic data, like the one presented in Figure 5A, was collected for nanotubes of different diameters. The average receding oxide rates (*m*_2_) were calculated from the linear part of the curve for each nanowhisker. Therefore, each data point in Figure 5B represents an average rate of the receding oxide – *m*_2_ of an individual nanowhisker during its conversion reaction into a WS_2_ nanotube. Notably, the rates differ significantly according to the reaction temperatures. Expectedly, at 900 °C, the rates are considerably smaller compared to those at 1000 °C. Importantly, the receding-oxide rate is strongly diameter-dependent at 900 °C. This behavior directly reflects the curvature-dependent nature of evaporation, where higher-curvature tips (smaller-diameter oxide cores) evaporate faster than those with larger diameter. The diameter dependence diminishes at 1000 °C, where the evaporation gets so fast that it becomes limited by the local gas equilibrium within the cavity. Nanowhiskers with small diameters tend to react promptly compared with their thicker counterparts. Deductively, at higher temperatures, the evaporation of the oxide is predominant, and the deposition of the WS_2_ layers from the vapor phase within the cavity is expected to be favored compared to the rate of gas–solid reaction i.e., the “surface-inward” mechanism.

Undoubtedly, there are two main pathways for the sulfidation reaction of tungsten oxide nanowhiskers. First is the “surface-inward” reaction pathway, which is rather fast in the initial reaction stage, ergo, the formation of the first outer WS_2_ walls. However, this reaction is indeed self-limiting due to a diffusion barrier effect of the growing WS_2_ layers. The environment of the reaction (W_18_O_49_ nanowhisker, H_2_S, H_2_ altogether at elevated temperatures), favors volatilization of the tungsten suboxide nanowhiskers, which are known to evaporate from its tips along the ⟨010⟩ direction of the crystal lattice due to its high surface energy. This process is initialized as soon as the reactants reach the reaction temperature (see Figure 3, 3 min), but the specific reaction rate is influenced also by the detailed structure of the precursor nanowhisker, like the nanowhisker diameter, as seen in Figures 2 and 3. The volatilized tungsten oxide reacts with H_2_S and H_2_ internalized in the core, depositing WS_2_ layers from the vapor phase within the core. The newly formed layers are deposited on the pre-existing ones within the cavity. This mechanism could be described as a “receding oxide core”. Both mechanisms take place coincidentally during the conversion of the oxide nanowhisker into a WS_2_ nanotube and are critical for its morphological outcome. First, the “surface-inward” mechanism is responsible for creating the outer shell formed by several WS_2_ layers. Then, the kinetics of the “surface inward” reaction is slowed down appreciably because the reactive H_2_S diffuses rather slowly through the basal surface of the already formed layers. The “receding oxide core” is becoming predominant the higher the reaction temperature is, completing the formation of the nanotube. The ratio between the rate of the two mechanisms is determined by a variety of parameters, including the temperature and the vapor pressure of the oxide within the nascent nanotube.

Finally, the ultimate bedrock is that the so-called “surface-inward” reaction mechanism is fundamentally the reaction between vapor (H_2_/H_2_S) and solid phase (W_18_O_49_ nanowhisker). On the other hand, the “receding oxide core” allows deposition of WS_2_ layers from the vapor phase (H_2_/H_2_S/volatile WO_2_(OH)_2_) mostly in the nanotube interior. Indeed, the kinetics of both processes changes based on chosen reaction parameters, growth environment, and selected precursors. The following text addresses some nuances of both mechanisms, with additional figures presented as a supplement.

Figure 5B indicates a strong temperature dependence of the formation kinetics. To gain more detailed insights into the balance between the mechanisms in play, a series of experiments were carried out at 800 °C. Figure S4 displays a series of *ex situ* TEM images showing the sulfidation process of a W_18_O_49_ nanowhisker at 800 °C, where the evaporation rate is appreciably slower than at 1000 °C. Despite that, and in analogy to the experiments carried out at 1000 °C (Figure 3), a cavity was formed near the tip of the nascent nanotube here, too. Like in the higher temperature reaction, the WS_2_ layers were formed at the interface with the oxide core. However, the tungsten oxide gradually diminished, both along the axis (direction marked by yellow arrow) and from the sides of the nascent nanotube (red arrows). Notably, comparing the oxide core at 0, 3, 10, and 30 min of the reaction shows a diminution of the oxide core diameter in steps from 22, 17, 13, and 9 nm, respectively. Since the oxide core is simultaneously receding and thinning, one can assume both “receding oxide core” and “surface-inward” mechanisms acted conjointly here.

*Ex situ* TEM analyses displayed in Figures 3 and S4 allowed a comparison of the deposition rate of the WS_2_ layers with time. Figure S5 describes the time dependence of the deposited layers at 800 and 1000 °C in the nascent nanotube’s tips. At the initial stage of the reactions (0–10 min), the number of layers in the tip is identical for both cases. However, as the oxide core recedes the layer deposition became temperature dependent. At 1000 °C (followed in Figure 3) additional WS_2_ layers are continually deposited from the vapor phase inside the lumen. On the other hand, at 800 °C (displayed in Figure S4) the evaporation rate of the oxide core is slower, and therefore new layers are not deposited in the tip as much as at 1000 °C. Most of the oxide vapor was converted into sulfide near the receding oxide front, and new WS_2_ layers were formed along the lumen (see Figure S4 at 30 and 720 min). In parallel to this reaction, the H_2_S and H_2_ reacted with the solid tungsten oxide core via the “surface-inward” mechanism.

Another critical factor for the pathway of the reaction is the integrity and the crystallinity of the passivating WS_2_ layers conformably coating the W_18_O_49_ nanowhisker. This situation is particularly relevant in the initial steps of the reaction. Figure S6 shows the nascent WS_2_ nanotube after 10, 30, and 720 min of the sulfidation reaction at 1000 °C. Initially (10 min), the structure of the walls is not uniform, and a number of defects are visible, mainly at the tip of the nanotube. The yellow arrow in Figure S6 marks a significant rupture in the nanotube tip. The presence of this defect allows the out-diffusion of the oxide vapor from the core and the diffusion of hydrogen and H_2_S into the cavity at the core. After an additional 20 min of sulfidation, these defects previously present at the tip are gradually healed by recrystallization and also WS_2_ deposition from the vapor phase (marked by yellow arrow). Consequently, the diffusion of the species both in and out of the core is somewhat hindered and becomes more sluggish. After 720 min, the nanotube had nearly perfectly aligned walls, and the defect was healed completely.

The current observations are also able to explain the occurrence of open-ended nanotubes within the experimental batches. In several other cases, the buildup of the inner vapor pressure of the volatile oxide in the core increased and punched the outer WS_2_ layers, usually in the cusps of the nanotube tip. Three instances of this phenomenon are illustrated in Figure S7A–C as TEM figures. Additionally, Video 4 provides an *in situ* SEM demonstration of this process (Video 4, time 3:12:14 to 3:16:48). In Figure S7A, the expansion of the gaseous tungsten oxide vapors blew up the cap, producing a bubble-like polyhedral shape outside the nanotube tip. In Figure S7B, the oxide vapor escaping through the nanotube tip produced (after reaction with H_2_S) nonoriented WS_2_ plates sticking randomly out at the tip. The last Figure S7C shows a nearly complete ripped-out cap, which is still somewhat attached to the nanotube walls. Similar observations were done previously^52,53^ but were not framed into a comprehensive growth model for the nanotubes. The discussion here can help explain why some of the WS_2_ nanotubes have opened tips, while others are closed.

The results and analysis thus far show that a revision in the well-accepted growth mechanism of the nanotubes is desirable. Early on, the growth mechanism of fullerene-like (quasi-spherical) nanoparticles of WS_2_ from WO_3_ nanoparticles via the “surface-inward” growth mechanism was investigated and confirmed.^50,51,54^ This model was extended and modified to the growth of WS_2_ nanotubes, which can be obtained directly from the WO_3–*x*_ nanoparticles or via intermediary production of W_18_O_49_ nanowhiskers and its subsequent sulfidation.^2,4,10^ The current work shows that in addition to the established “surface inward” growth model another mechanism for converting W_18_O_49_ nanowhiskers into multiwall WS_2_ nanotubes operates. Some hints to this growth model were obtained in the past^52−55^ but were not systematically investigated. The receding oxide core and the conformal growth of WS_2_ within the cavity in the oxide core were visible but did not materialize into a firm growth mechanism, which is designated now as the “receding oxide core” model.

The critical question is whether the above-mentioned mechanisms have implications for the WS_2_ nanotube synthesis in the flow reactors. To address this issue, two sulfidation reactions in the flow reactor were interrupted after 10 and 30 min of the reaction time, respectively. The TEM analysis of the reaction outcome after 10 min (Figure S8A) and 30 min (Figure S8B) is a representative example of a multitude of nascent WS_2_ nanotubes from the flow reactor. Complementary TEM images of the same batch are also displayed in Figure S9. In Figure S8A the W_18_O_49_ nanowhisker is covered with 3–5 WS_2_ layers after 10 min of the sulfidation reaction, similar to those synthesized within μReactor. The tip of the emerging WS_2_ nanotube is already hollow with visible ruptures from gaseous tungsten oxide release (see yellow arrows). The inner tungsten oxide core shows a distinct meniscus analogous to the one observed in the nanotube produced in the μReactor (see, for comparison, Figures 3). In analogy to the products obtained in the μReactor (Figure 3), some WS_2_ layers were also deposited on the outside surface of the nanotube (see the red arrow). Comparably to Figure 4C, WS_2_ layers were also formed in the cavity of the core near the tip of the nanotube. After 30 min sulfidation, the hollow core of the different nanotubes became substantially larger (see Figure S8B), and many more WS_2_ layers have accumulated on the nanotube walls (approximately 15 layers). However, since the thickness of the wall is uniform in the cavity and across the oxide-sulfide interface, it is clear that both the “receding oxide core” mechanism and the well-established “surface-inward” mechanism do occur conjointly. The oxide core receded from the tip along the ⟨010⟩ axis, forming ever larger cavities from the ends of the nascent nanotube yet preserving the meniscus. Therefore, it is possible to state that the “receding oxide core” mechanism for the formation of WS_2_ nanotubes coexist together with the known “surface-inward” mechanism in the flow reactor. Importantly, the current mechanistic insight explains the nascency of the various WS_2_ nanotube morphologies observed throughout the samples and described in the literature. One can explain the existence of nanotubes with several WS_2_ layers and significant cavities.^36,56−58^ In such cases, the oxide core evaporated from the nascent nanotube, leaving an empty volume in its wake.

## Conclusions

In the present work, a modified μReactor suitable for sulfidation reactions *in situ* in the SEM is introduced. The heated reaction-module (MEMS chip) can be transferred to the TEM for the high-resolution examination of a specific nanostructure and back to the SEM for resuming the reaction and so forth. The contemporary insight was gained into the growth mechanism of WS_2_ nanotubes from W_18_O_49_ nanowhiskers using the μReactor and *ex situ* TEM analyses of the sulfidation reaction. The mechanism of the synthesis of the WS_2_ nanotubes has been profoundly studied in the past. However, the exact step-by-step reaction pathway was unknown until now. The nascency of the WS_2_ nanotube starts with swift reaction of the H_2_S/H_2_ gas mixture with the reactive tungsten suboxide surface. This reaction leads to the formation of several passivating layers of WS_2_ of the oxide nanowhisker surface through the well-established “surface-inward” mechanism. This initial shell preserves the nanotube’s outer contour and slows further reaction between the solid oxide and the gaseous H_2_S. Conjointly with the slow surface-inward solid–gas reaction, the inner oxide core reacts with in-diffusing H_2_ gas and is anisotropically volatilized in the ⟨010⟩ direction forming a cavity of the nascent nanotube. The reaction progresses inward, forming a growing cavity in the core. The pressure inside the core of the nascent nanotube builds up during this process, and the gas leaks through the weak (defective) spots in the initial WS_2_ shell. The out-diffusion of the tungsten oxide vapors violates the tungsten stoichiometry between the initial oxide nanowhisker and the nanotube product. Simultaneously, the oxide vapors react with the in-diffusing H_2_S, resulting in WS_2_ molecules which are deposited as a new layer on the preexisting WS_2_ shell inside the cavity. This reaction mechanism, which was not discussed earlier, is named the “receding oxide core”. The spectrum of experimental parameters, like gradients in the gas pressure between the SEM chamber and the μReactor, and the μReactor and inner core of the nanotube, temperature, diameter of the oxide nanowhisker, etc., influences the morphology of the nanotubes and is discussed extensively in the text. The kinetics of the receding oxide core and cavity growth are studied quantitatively. The receding oxide core exhibits two kinetic stages: Initially (near the tip), the rate is followed by a slower process, as the oxide core recedes toward the center of the nanotube. As complementary experiments, the growth of nanotubes in the atmospheric pressure flow reactor is carried out as well. These experiments show that the proposed growth model is valid also under regular reaction parameters in addition to the already established surface-inward mechanism.

The upgraded μReactor for the *in situ* growth in SEM coupled with the *ex situ* sequential TEM analysis proved to be a vital and emergent platform for studying high-temperature heterogeneous reactions of individual nanostructures.

## Methods/Experimental

The W_18_O_49_ nanowhiskers were prepared according to the previously reported protocol.^44^ W_18_O_49_ nanowhiskers were dispersed and ultrasonicated in isopropyl alcohol (pure, PENTA, Czech Republic), forming a transparent, blue suspension for drop casting on microelectromechanical system (MEMS) chips. Hydrogen sulfide (99.5%, Linde, Czech Republic) and hydrogen (99.999%, Linde, Czech Republic) gases were purchased in 2.5 l and 10 l cylinders and used as received.

### Modified SEM Fitted with μReactor Dedicated to Sulfidation Reactions

**Figure 6:**
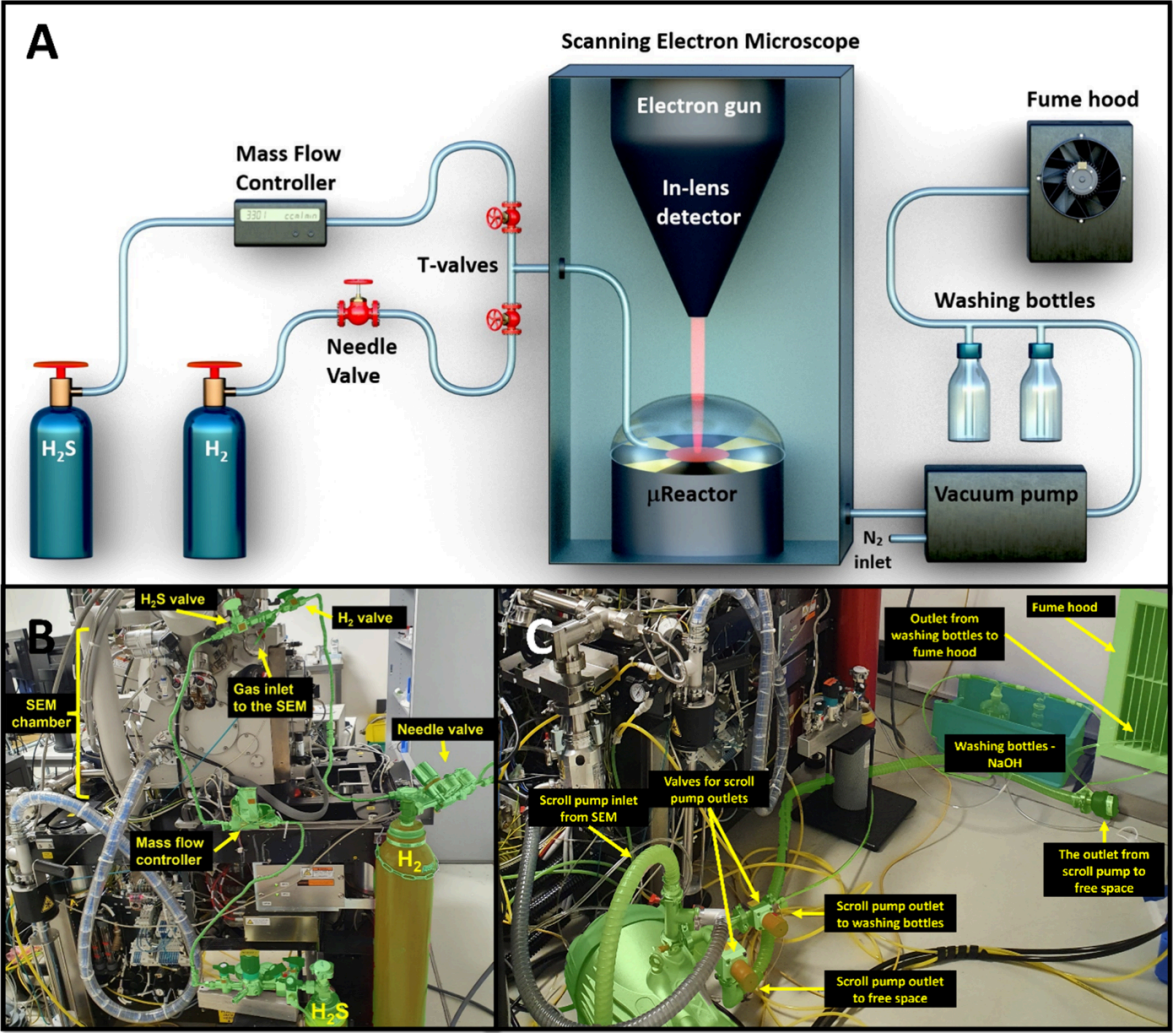
Schematic rendering (A) of the modified SEM with an μReactor dedicated to studying sulfidation reactions at elevated temperatures. The utilized gases are hydrogen sulfide and hydrogen, which are fed through the piping and valves’ system. A mass-flow controller and needle valve are used to control the H_2_S and H_2_ supplies, respectively. The μReactor consists of a reactor body, a heating MEMS chip with a sample, and a lid for maintaining the gas pressure. The canopy of the reactor has a gas inlet and an aperture at the top for the incident electron beam and for collecting the backscattered electrons (BSE). The sulfidation reaction is inspected *in situ* via an in-lens BSE detector T1. Notably, the μReactor is pressurized up to 500 Pa (5 mbar), while a system of vacuum pumps keeps the SEM chamber under a high vacuum. The final gas outlet is directed to washing bottles with sodium hydroxide solution for H_2_S scrubbing and subsequently to the fume hood. Schematized parts are visualized in photographs (B) and (C), showing the gas inlet and outlet of the SEM, respectively.

A dedicated scanning electron microscope (SEM) was used for these experiments (Thermo Fisher Scientific Scios DualBeam FIB-SEM). Schematic drawing of the modified μReactor is displayed in Figure 6A, and a photograph showing the gas system inlet and outlet is shown in Figure 6BC.

The heating in the μReactor is secured by utilizing a microelectromechanical (MEMS) chip.^59^ The advantage of the MEMS chip is its compatibility with TEM due to the electron-beam-transparent silicon nitride membrane on which the sample is placed. Therefore, the current SEM setup allows for *in situ* SEM sulfidation and *ex situ* TEM analyses of the same sample.

### *In Situ* SEM Analysis of Continual Sulfidation of W_18_O_49_ Nanowhiskers in the μReactor

The sulfidation experiments were performed in a specifically modified scanning electron microscope Scios (Thermo Fisher Scientific Scios DualBeam FIB-SEM) equipped with an μReactor (Figure S10) utilizing a MEMS chip for sample heating. A detailed description of the μReactor within the SEM was presented elsewhere.^44^ The H_2_S and H_2_ gas inlets were designed as a T-shaped piping valve system equipped with a mass flow controller and a needle valve, respectively (Figure 6A). The mixed gases flow into the μReactor, surrounding the sample placed on the MEMS heating chip and then escaping into the SEM chamber through the hole in the imaging aperture. The standard vacuum system of the SEM continuously pumps the gases. The outlet of the scroll pump is directed into the dual gas washing bottles with an alkaline solution for the chemical neutralization of the H_2_S gas (Figure 6B). The final gas outlet was located in the fume hood.

The usual *in situ* sulfidation procedure of W_18_O_49_ nanowhiskers was performed as follows. The W_18_O_49_ nanowhisker sample dispersed in isopropanol was drop-casted on a MEMS chip and mounted in the μReactor body. The SEM chamber was evacuated, and the nanowhiskers sample was localized on the chip. For all SEM image acquisitions, the acceleration voltage was set to 20 kV and the current was set to 0.8 nA. The dose rate was calculated to be 300 *n*_e-_·nm^–2^·s^–1^ (number of electrons per square nanometer per second). The electron beam effect was studied in a previous work^44^ demonstrating additional reductive force and heating of the sample. Therefore, the reaction is influenced by the observation. However, *ex situ* TEM analysis was performed without an SEM observation to limit the effect of the electron beam. For complete picture regarding the reaction mechanism, reactions in flow reactor were performed. Subsequently, the μReactor was closed and pressurized by hydrogen (25 Pa) and hydrogen sulfide (50 Pa). The visualization of the sample was accomplished through the aperture in the pressure-limiting lid using an in-lens detector (T1 detector, Scios). The sample was heated to the reaction temperature (800 or 1000 °C, at a heating rate of up to 1000 K/s). Since the aperture of the reactor canopy is very small, the BSE signal is very weak, and therefore, the acquisition time is rather long. The typical acquisition time for a frame was between 600 and 3000 s. The overall analysis was performed over multiple hours up to 1 week due to the low reaction rate. The reaction was terminated by rapid cooling down the MEMS chip. The image sequences collected by the SEM were processed into drift-compensated videos. The individual frames were also processed by ImageJ software.

### *Ex Situ* TEM Sequential Analysis of the W_18_O_49_ Nanowhiskers Sulfidated in the μReactor within SEM

Repeated *ex situ* analysis of individual nanowhiskers in the TEM was enabled thanks to the MEMS chip (Figure S11A), which was compatible with both kinds of microscopes and could be easily switched between the two. The MEMS chip consisted of a silicon nitride membrane transparent for the TEM e-beam (Figure S11B). Likewise, the *in situ* SEM and sulfidation for the subsequent *ex situ* TEM measurements were performed in the μReactor within SEM. While the SEM sequence was measured continually and by the same SEM in which the reaction was performed, the TEM analysis had to be done *ex situ* periodically at preselected reaction times.

The TEM analysis was performed using a Thermo Fisher Scientific Talos F200i instrument operated in HRTEM mode at a high voltage of 200 kV and beam current of 1 nA. TEM images were postprocessed in the Velox and ImageJ software. Initially, the W_18_O_49_ nanowhiskers were drop-casted on the MEMS chip and observed in the SEM to ensure the proper sample placement on the transparent membrane. Following that, TEM images at multiple magnifications were acquired from selected W_18_O_49_ nanowhiskers on the chip using the Thermo Fisher Scientific NanoEx-i/v holder (Figure S11C). The MEMS chip with the sample was then placed into the μReactor in the SEM. Sulfidation was performed at 1000 °C in H_2_/H_2_S mixture (25 + 50 Pa, respectively) for 720 min. TEM measurements were carried out intermittently after 3, 10, and 30 min of reaction time. In every interruptive TEM measurement, the heating was stopped, and the SEM chamber with the gas inlets was left to evacuate for at least 1 h. The MEMS chip was carefully extracted, and the specimen was analyzed in TEM. Subsequently, the MEMS chip was placed back into the μReactor again, and the sulfidation reaction continued. Engaging this procedure, sequences of TEM images of the same W_18_O_49_ nanowhiskers transforming into the WS_2_ nanotubes were acquired.

### Sulfidation in the Atmospheric Pressure Flow Reactor

For comparison between the μReactor and a large-scale atmospheric pressure synthesis, several milligrams of W_18_O_49_ nanowhiskers prepared according to a previously reported protocol^44^ were subjected to high temperature (845 °C) reductive sulfidation in a stream of a mixture of H_2_S (7%) and H_2_ (3.5%) in nitrogen (total flow 150 mL.min^–1^). The reaction was performed for 10 and 30 min, after which the samples were swiftly extracted from the hot zone in the horizontal quartz tube flow reactor. The collected materials were drop-cast onto TEM grids and analyzed by HRTEM.

## Supporting Information Available

*In situ* scanning electron microscopy and *ex situ* transmission electron microscopy analyses of the growth of WS_2_ nanotubes (Figures S1–S9); schematic rendering and photos of the μReactor used for *in situ* SEM growth of WS_2_ nanotubes (Figure S10); schematics and photos of the MEMS chip used for the *ex situ* TEM analysis (Figure S11) (PDF)
Videos 1–4 on growth mechanism, *in situ* sulfidation reaction, and *in situ* SEM process (ZIP)

## Dedication

This article is dedicated to CNR Rao, a mentor and close friend, for his 90th birthday. We wish him many years of health, fruitful research, and numerous more contributions to the scientific landscape of India.
