# Supplementary material for: Mechanism of WS2 Nanotube Formation Revealed by in Situ/ex Situ Imaging
Source: ACS Nano. 2024 May 3;18(19):12284–94. doi: 10.1021/acsnano.4c01150 (PMC11100282; doi:10.1021/acsnano.4c01150)
Supplement: Supplementary file 1 — nn4c01150_si_001.pdf [file nn4c01150_si_001.pdf]

Supplementary material for:

Mechanism of WS_2 Nanotube Formation Revealed by *in Situ/ex Situ* Imaging

Vojtěch Kundrát,[ab]* Libor Novák,[b] Kristýna Bukvišová,[bc] Jakub Zálešák,[bd] Eva Kolíbalová,[c] Rita Rosentsveig,[a] M.B. Sreedhara,[e] Hila Shalom,[f] Lena Yadgarov,[f] Alla Zak,[g] Miroslav Kolíbal,[ch] Reshef Tenne[a]*

[a] Department of Molecular Chemistry and Materials Science, Weizmann Institute of Science, Rehovot 7610001, Israel

[b] Thermo Fisher Scientific, Vlastimila Pecha 12, 62700 Brno, Czech Republic

[c] Central European Institute of Technology, Brno University of Technology, Purkyňova 123, 61200 Brno, Czech Republic

[d] Chemistry and Physics of Materials, University of Salzburg, Jakob-Haringer-Strasse 2A, 5020, Salzburg, Austria

[e] Solid State and Structural Chemistry Unit, Indian Institute of Science, CV Raman Rd., Bangalore, 560012, India

[f] Department of Chemical Engineering, Ariel University, Ariel 4070814, Israel

[g] Faculty of Science, Holon Institute of Technology, Golomb Street 52, Holon 5810201, Israel

[h] Institute of Physical Engineering, Faculty of Mechanical Engineering, Brno University of Technology, Technická 2, 616 69 Brno, Czech Republic

*Corresponding author(s)

Email: reshef.tenne@weizmann.ac.il; vojtech.kundrat@weizmann.ac.il

KEYWORDS: WS_2 nanotube, sulfidation, in-situ, ex-situ, electron microscopy, reaction mechanism

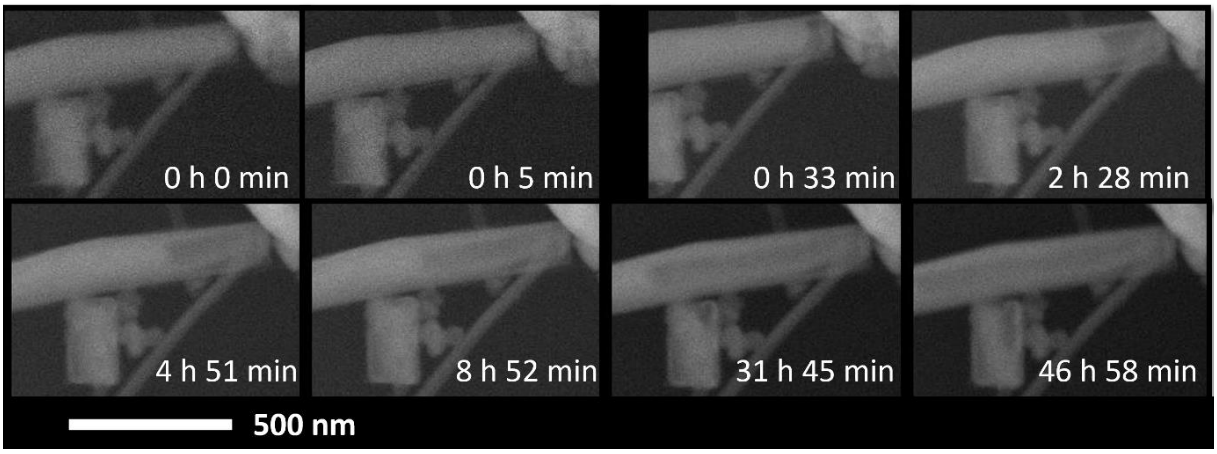

Figure S1. Collection of SEM images from *in situ* sulfidation reaction of tungsten oxide nanowhiskers at 800 °C in a mixture of 50 Pa of H_2S and 25 Pa of H_2. The cavity formation and the nascent nanotube at the tip is similar to the one in Figure 2.

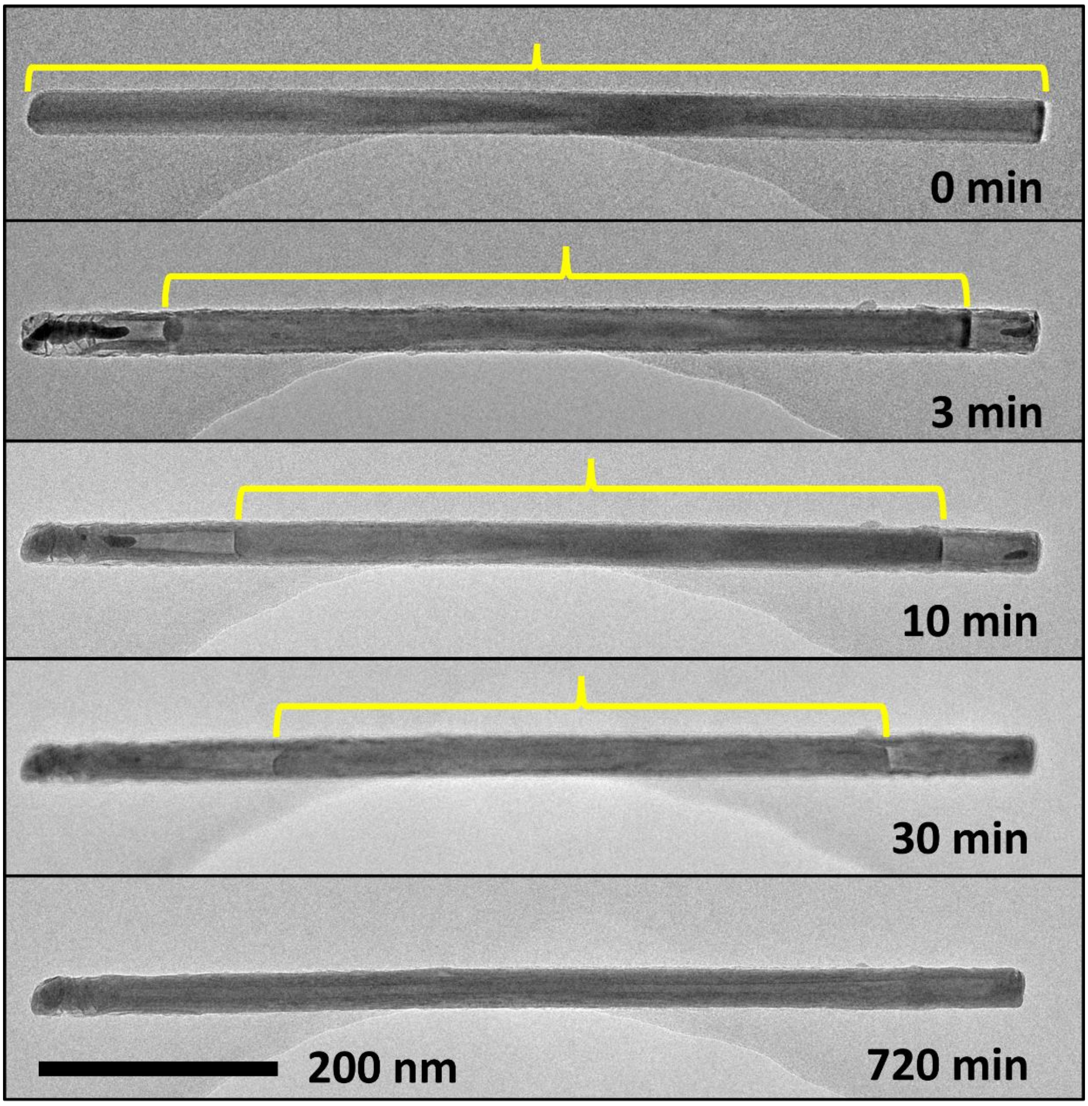

Figure S2. *Ex situ* observed progress of WS_2 nanotube formation from $W_{18}O_{49}$ nannowhisker. Cavities are formed from both ends of the nascent nanotube. Interestingly, at 3 and 10 minutes darker tungsten oxide species were precipitated within the nanotube cavities due to high cooling rate of the chip. Furtherly they react with H_2S and H_2, incorporating into the WS_2 nanotube walls. Yellow braces mark the size of the tungsten oxide core in time.

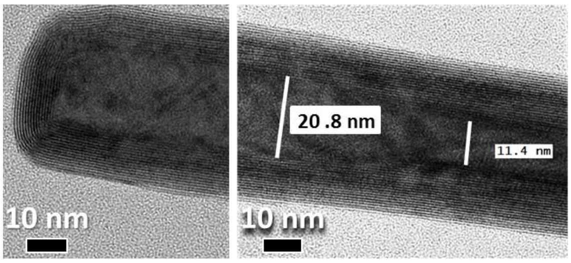

Figure S3. TEM image of a WS_2 nanotube prepared in the atmospheric flow reactor at 845 °C with a larger cavity near its tip and a narrow lumen in the middle

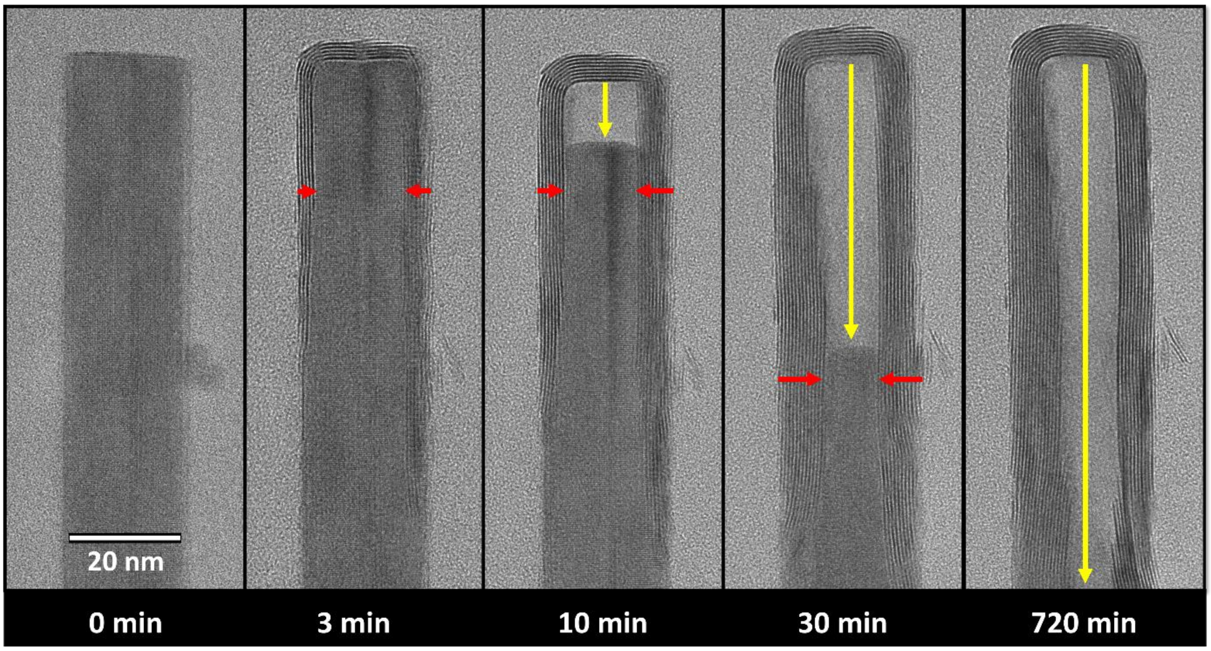

Figure S4. Additional sequence of *ex situ* TEM images of nanotubes prepared at 800 °C. Initially (3 min), a passivation layer consisting of a few WS_2 walls is formed, enveloping mainly the tip of the nanowhisker. Longer sulfidation leads to the evaporation of the oxide core, which recedes from the tip, forming new WS_2 layers within the core of the nascent nanotube. Note the diminishing of the oxide core both from the tip and from its sides, showing together "receding oxide core" and "surface-inwards" mechanisms.

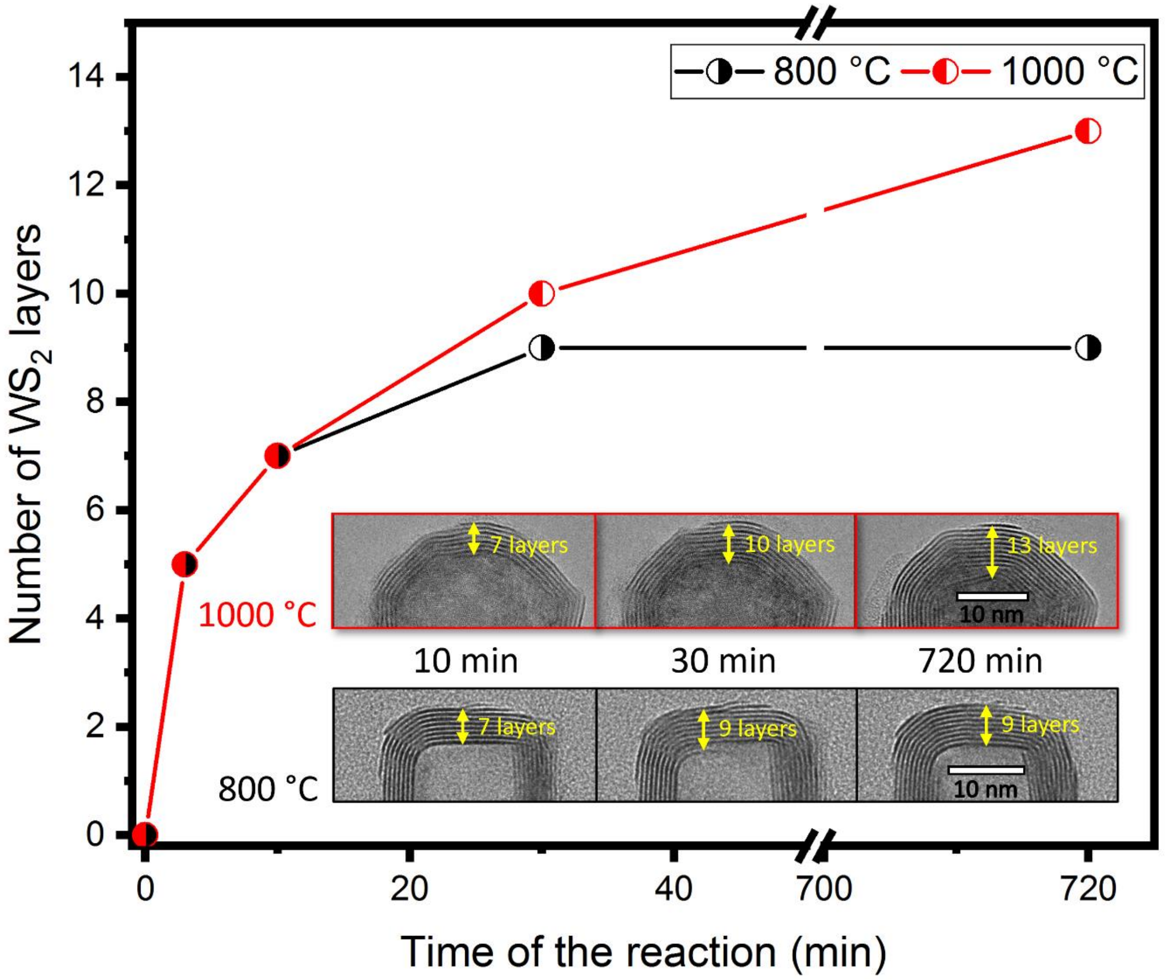

Figure S5. Time dependence of the number of WS_2 layers in the nascent nanotube tip formed at 800 (black) and 1000 °C (red) and described *ex situ* TEM and displayed in Figure S3 (800 °C) and 3 (1000 °C). At 1000 °C, new layers are formed within the tip even due to sufficient evaporation of the oxide core in the cavity. At 800 °C, the evaporation of the oxide core is not so significant. Therefore, new layers in the tip are not deposited at a high rate.

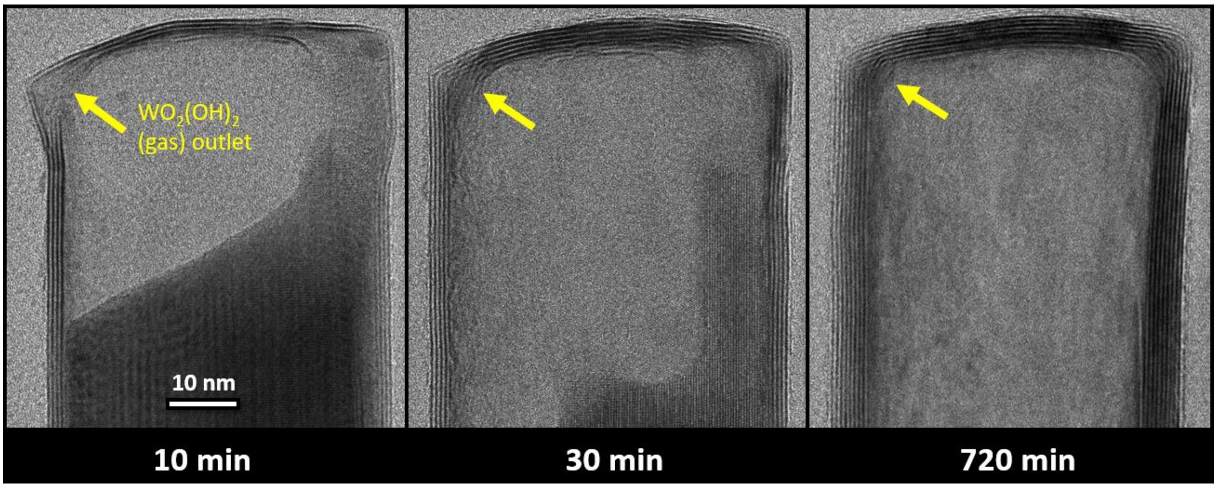

Figure S6. The self-healing process of a WS_2 nanotube during its synthesis at 1000 °C is followed *ex situ* by TEM. Sequential analysis of the nascent WS_2 nanotube with $W_{18}O_{49}$ core is shown. Interestingly, at 10 minutes of reaction time, a rupture in the WS_2 layers is observed (yellow arrow). As the reaction proceeds (30 and finally 720 minutes), the rupture is self-healed by conformal deposition of additional WS_2 layers within the core.

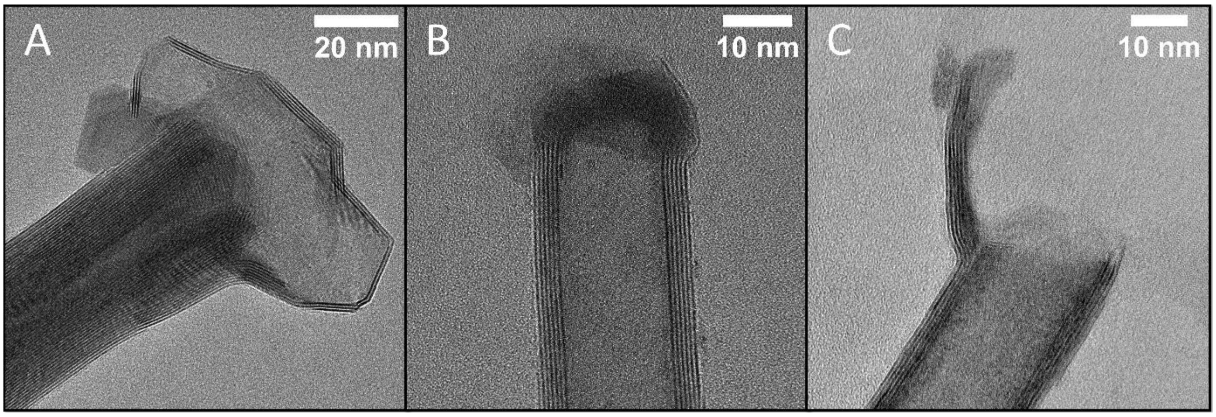

Figure S7. TEM analyses of nanotube tips with partially removed nanotube cap due to rapid expansion of vaporized tungsten oxide core. Acquired TEM images are from different sulfidation experiments in the μReactor (A&B) and atmospheric flow reactor (C)

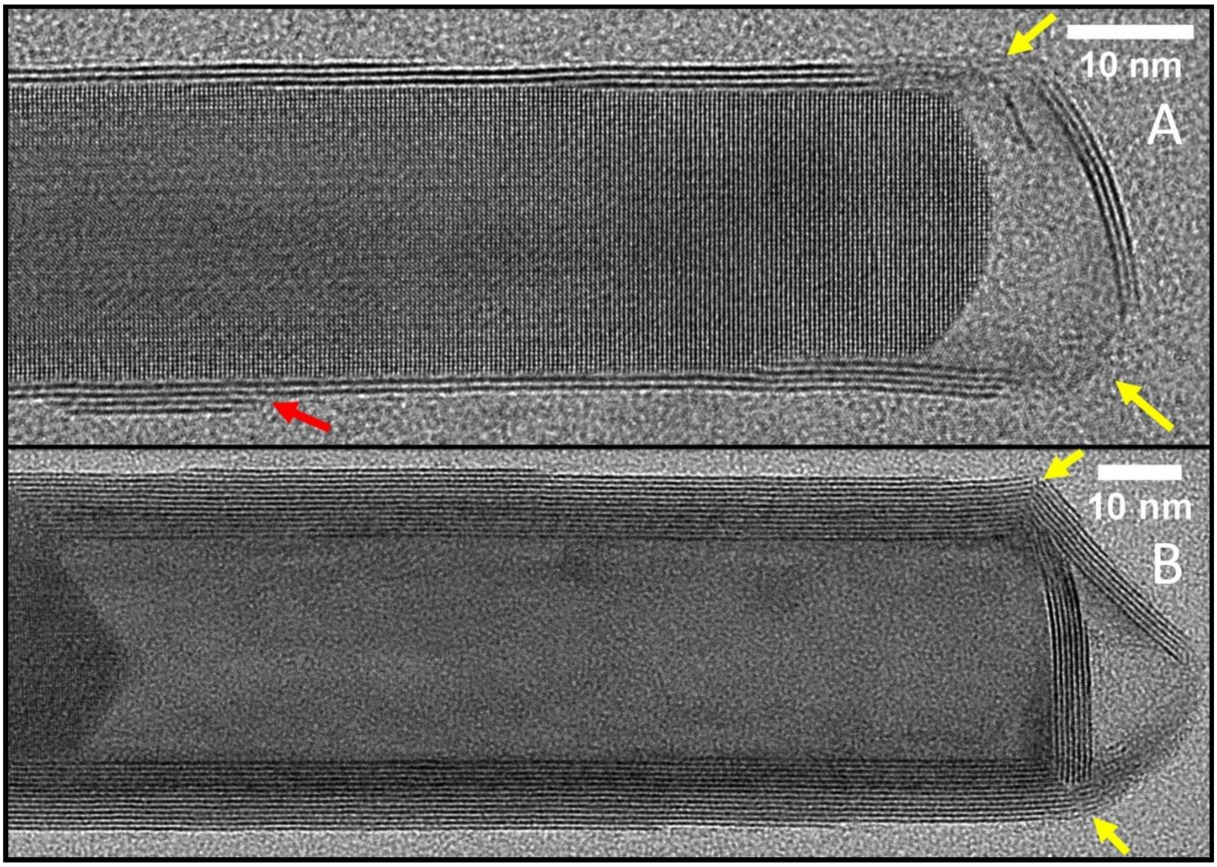

Figure S8. TEM analysis of nascent WS_2 nanotubes formed in the atmospheric pressure flow reactor at 845 °C (detailed experimental parameters described in the Experimental section of the ms.). The specimen (A) is a $W_{18}O_{49}$ nanowhisker covered by several Ws_2 layers following a swift extraction of the oxide precursor from the hot zone after 10 min of reaction time. The meniscus of the oxide core and the forming nanotube cavity are observed at the tip of the nascent nanotube. The yellow arrows mark the defects through which oxide vapor effused out of the core (and H_2S and H_2 diffused in). This pattern is similar to protrusions observed in nanotubes prepared in the μReactor. The red arrow points to the deposited WS_2 layers on the outside of the nascent nanotube. (B) shows another nascent WS_2 nanotube after 30 minutes of reaction time. Clearly, the number of WS_2 layers is larger than in (A), and the nanotube cavity is also larger. The nanotube has a distinct cap with ruptures marked by yellow arrows, which allow oxide vapor to effuse out of the core.

Two reactions in the horizontal flow reactor were performed and disrupted after 10 and 30 minutes for comparison with the low-pressure reactions in the μReactor. The samples were analyzed by HRTEM, as displayed in Figures S7 and S8. Figure S8A shows the nascent nanotube with the tungsten oxide core after 10 minutes of the reaction. Indeed, in analogy to the reactions carried out in the μReactor, a cavity was formed in the oxide core at the tip of the nanotube here, too. Furthermore, the tip of the nanotube is oval, and the oxide has a convex meniscus, as observed in the nanotube displayed in Figure 3. The morphology of the tip in Figure S8B is somewhat similar, but its shape is more faceted. Figure S8C is nearly identical to that of Figure S8B. Interestingly, however, a flake near its tip was possibly ripped out from the structure. The nascent nanotube in Figure S8D follows closely again the formation pathway described in detail throughout the work. Figures S8E and S8F show new layers growing directly from the oxide core. Indeed, a similar structure was already observed in Figure 4A. Such growth is promoted by the high inlet of the reactive H_2S and H_2 together with a relatively low volatilization rate of the oxide core. Figure S8G shows the core of the nascent WS_2 nanotube after 30 minutes of reaction. Again, the core is diminishing from the nanotube end. In Figure S8H, the cavity of the nanotube is relatively large. Finally, Figures S8J and S8K show lower magnification TEM micrographs of nanotubes with a cavity at their tips. Based on the comparative reaction in the flow reactor and the following analysis, it is evident that the "receding oxide core" mechanism of the WS_2 nanotube growth is valid for both the low-pressure μReactor and atmospheric-pressure reactor under the specific conditions used here.

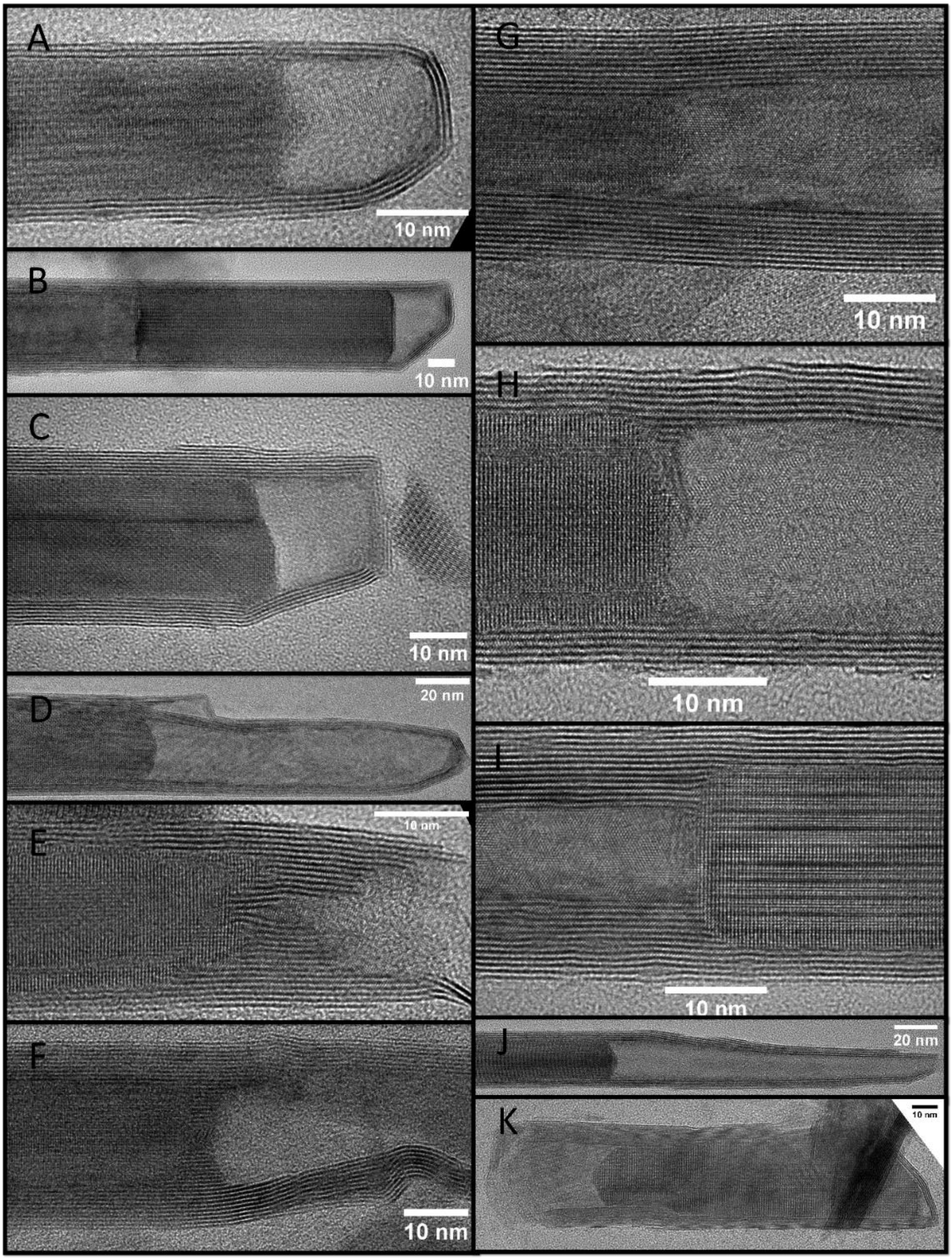

Figure S9. TEM analysis of various nascent nanotubes taken after 10 (A-F) and 30 (G-K) minutes of reaction in the atmospheric horizontal flow reactor at 845 °C (detailed experimental parameters described in the Experimental section of the ms.). In all observed cases, the nanotube cavity was formed from the ends and followed the proposed vapor phase mechanism. The individual figures are described in the corresponding text.

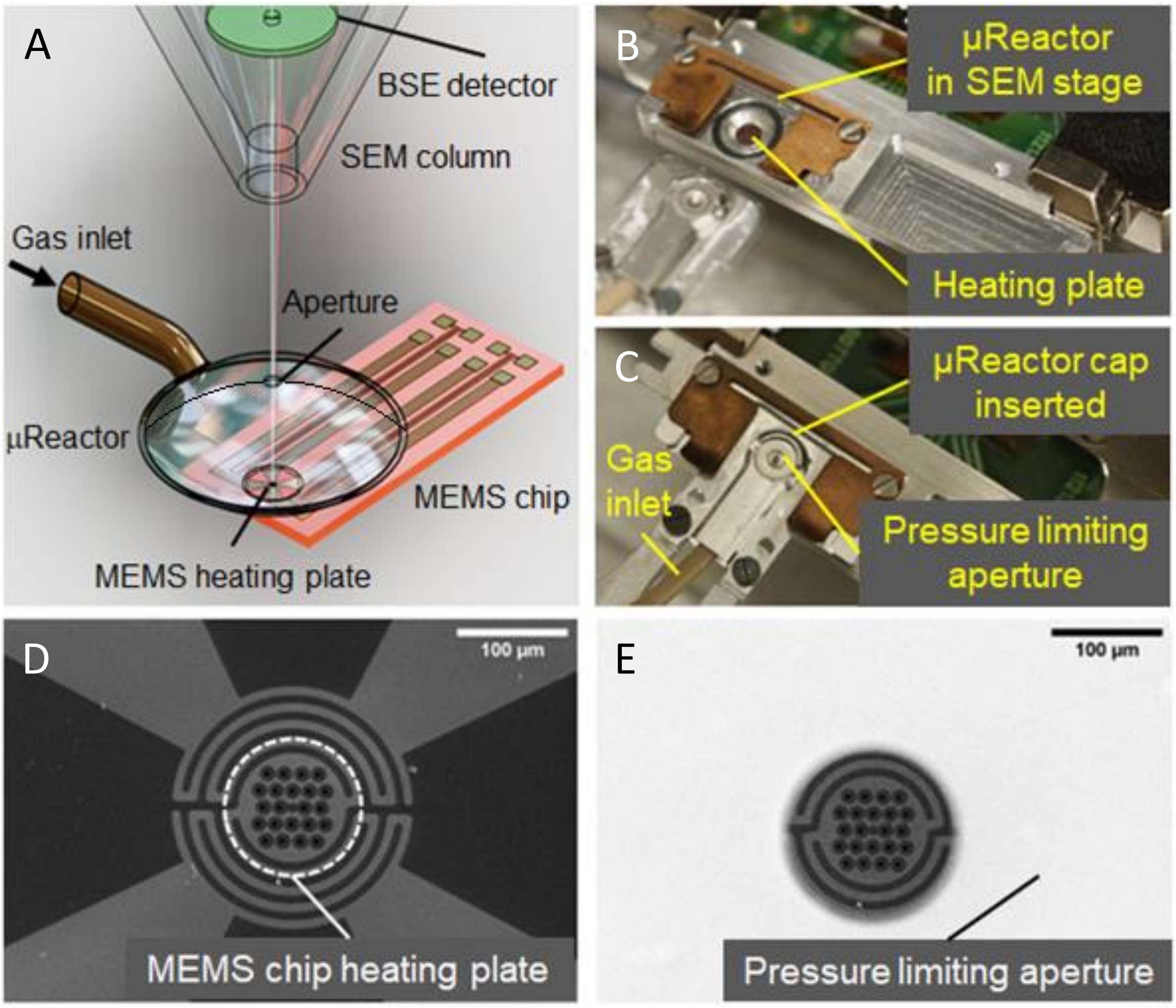

Figure S10. (A) Schematic rendering of the µReactor in the SEM. (B) photo of the µReactor with a retractable cap. (C) photo of the inserted µReactor tightened and closed by the insertion cap. (D) SEM image of the MEMS heating plate. (E) SEM image of the MEMS heating plate with the closed cap. The reaction volume is open during sample placement on the heating plate (B, D). When a sample is set in place and aligned under the electron beam (e-beam), the cap of the µReactor is inserted and tightened (C). The reaction volume is thus closed and sealed. The pressure-limiting aperture assures overpressure inside the reactor (up to 500 Pa) in the cap viewed by the SEM from the top (white area in (E)). Note that the SEM chamber remains under high vacuum conditions. *In situ* SEM imaging is possible through the hole in the aperture (E). A gas inlet is incorporated into the cap. The gas is evacuated from the µReactor mainly through the aperture. The reactor can be opened/closed inside the SEM chamber without the need for chamber venting, which assures clean sample preparation (by FIB) and sample positioning (by the manipulator needle) without exposure to air. The sample deposited on the heating segment of the MEMS chip reacted with the admitted gas or under vacuum at elevated temperatures.

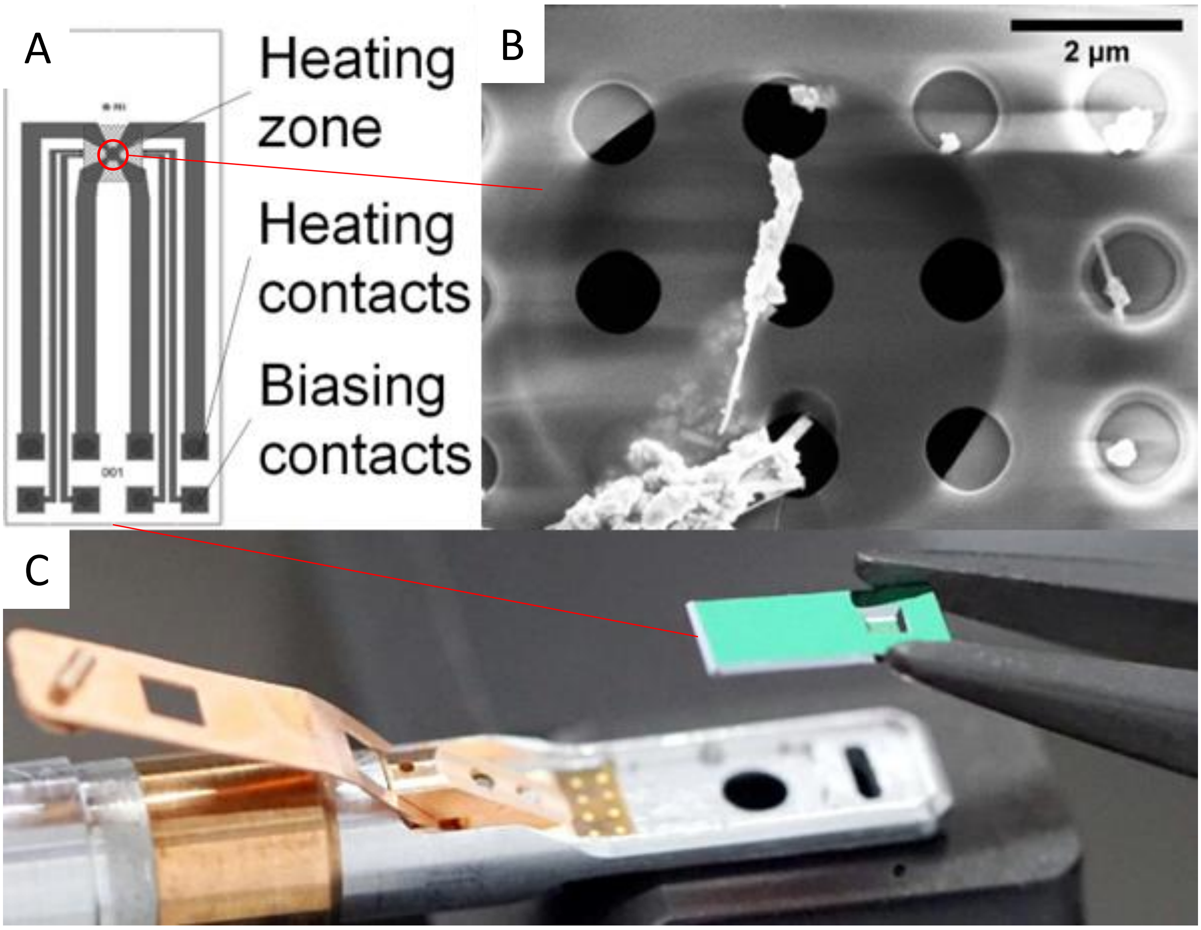

Figure S11. (A) Scheme of the MEMS chip (green rectangular object in the tweezers in C) used for the *ex situ* TEM experiments; B) SiC membrane on the MEMS heating chip; C) Thermo Fischer Scientific NanoEx-i/v heating and biasing holder for *in situ* SEM/TEM. The heating zone consists of multiple circular areas where the SiC membrane is thinned and perforated (B), allowing *ex situ* TEM measurements.
